# Bifunctional Oxygen Electrocatalyst of Mesoporous Ni/NiO Nanosheets for Flexible Rechargeable Zn–Air Batteries

**DOI:** 10.1007/s40820-020-0406-6

**Published:** 2020-03-09

**Authors:** Peitao Liu, Jiaqi Ran, Baorui Xia, Shibo Xi, Daqiang Gao, John Wang

**Affiliations:** 1 Key Laboratory for Magnetism and Magnetic Materials of MOE, Key Laboratory of Special Function Materials and Structure Design of MOE, Lanzhou University, Lanzhou, 730000 People’s Republic of China; 2 Institute of Chemical and Engineering Sciences, A*STAR, 1 Pesek Road, Jurong Island, 627833 Singapore; 3 Department of Material Science and Engineering, National University of Singapore, Engineering Drive 3, Singapore, 117575 Singapore

**Keywords:** Porous Ni/NiO, Oxygen reduction reaction, Oxygen evolution reaction, Electrocatalysis, Flexible Zn–air battery

## Abstract

One approach to accelerate the stagnant kinetics of both the oxygen reduction and evolution reactions (ORR/OER) is to develop a rationally designed multiphase nanocomposite, where the functions arising from each of the constituent phases, their interfaces, and the overall structure are properly controlled. Herein, we successfully synthesized an oxygen electrocatalyst consisting of Ni nanoparticles purposely interpenetrated into mesoporous NiO nanosheets (porous Ni/NiO). Benefiting from the contributions of the Ni and NiO phases, the well-established pore channels for charge transport at the interface between the phases, and the enhanced conductivity due to oxygen-deficiency at the pore edges, the porous Ni/NiO nanosheets show a potential of 1.49 V (10 mA cm^−2^) for the OER and a half-wave potential of 0.76 V for the ORR, outperforming their noble metal counterparts. More significantly, a Zn–air battery employing the porous Ni/NiO nanosheets exhibits an initial charging–discharging voltage gap of 0.83 V (2 mA cm^−2^), specific capacity of 853 mAh g_Zn_^−1^ at 20 mA cm^−2^, and long-time cycling stability (120 h). In addition, the porous Ni/NiO-based solid-like Zn–air battery shows excellent electrochemical performance and flexibility, illustrating its great potential as a next-generation rechargeable power source for flexible electronics.

**Electronic supplementary material:**

The online version of this article (10.1007/s40820-020-0406-6) contains supplementary material, which is available to authorized users.

## Highlights

- An oxygen electrocatalyst consisting of Ni nanoparticles interpenetrated in porous NiO nanosheets was successfully synthesized.
- The liquid Zn–air battery reveals a large open-circuit potential of 1.47 V, the maximum power density at 225 mW cm^−2^, and excellent device stability of over 120 h.
- The flexible solid-like rechargeable Zn–air battery shows excellent stability (no evident weakening after 240 cycles) and bendability.

**Electronic supplementary material:**

The online version of this article (10.1007/s40820-020-0406-6) contains supplementary material, which is available to authorized users.

## Graphical abstract

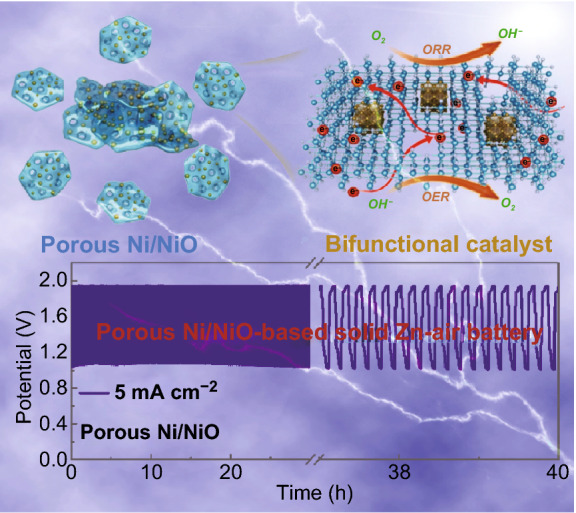

## Introduction

To meet the increasing demand for eco-friendly, renewable, and portable/mobile power sources, researchers have continued to study new energy conversion and storage systems [1–5]. Zn–air batteries have been widely studied owing to their high theoretical energy density, excellent safety, and eco-friendliness compared to other types of rechargeable batteries [6–9]. To improve the performance of Zn–air batteries, oxygen reduction and evolution reaction (ORR/OER) processes are required, where these reactions influence the charging–discharging progress [10–12]. Although Pt/Ir-based catalysts can perform well for either the ORR or OER, their application prospect is limited owing to the high cost and instability of these catalysts in commercial applications [13, 14]. Hence, there is an apparent urgency for exploring highly efficient non-noble metal electrocatalysts with rapid ORR/OER kinetics and superior overall performance.

Transition metals such as MnO_2_ [15], Co_3_O_4_ [16], and NiO [17] are considered as substitutes for noble metal-based electrocatalysts and have been in the spotlight as oxygen electrocatalysts for both the OER and ORR. For example, Zhuang and co-workers reported that the OER activity of transition metal oxides followed the order: NiO_*x*_ > CoO_*x*_ > FeO_*x*_ > MnO_*x*_, suggesting that NiO_*x*_ is an excellent candidate as an electrocatalyst [18]. However, most of these individual metallic oxides only afford a few of the desired ORR/OER performance parameters, rather than meeting the overall performance criteria. In addition, almost all of these oxides exhibit inherently poor electronic conductivity, which is a key problem that limits their electrocatalytic activity. Based on these understandings, considerable efforts have been devoted to developing solid-solution-type metal oxides, such as Fe-doped NiO and Ni-doped Co_3_O_4_ [19, 20], and nanocomposites of metal oxides/non-oxides, such as Fe–N–C nanosheet/NiO and NiO/Ni_2_P [21, 22]. A further approach is the use of conductive agents, such as carbon-based materials, which are incorporated to enhance the electron transfer to improve the OER activity [23, 24]. Nevertheless, the nanocomposite-type structure has to be controlled carefully to achieve synergy of the constituent components and properly control their interfaces. Otherwise, the overall performance will not be maximized, and the mechanical integrity will be poor, leading to peeling during long-term electrochemical cycling [25].

Indeed, the interface plays a critical role in enhancing the electrocatalytic performance of nanocomposite-type catalysts, where the heterojunction and fine-tuning of the interfacial electronic structure can be exploited. For example, tuning of the metal–semiconductor interface can effectively enhance the charge transport, as reported recently for graphene/Ni/MnO particles [25], Co/Ni_3_N nanowires [26], *N*-doped carbon/Co/CeO_2_ [27], and MoB/g-C_3_N_4_ nanosheets [28]. In electrocatalysts comprising Ni/NiO core@shell nanosheets, the metallic Ni core can accelerate electron transfer during the electrocatalysis process, while the amorphous NiO shell can lower the overpotential [21, 29, 30]. Gong and co-workers have proposed that the Ni/NiO interfaces are the active sites for HER catalysis in alkaline media [31]. More recently, a Ni–NiO heterostructure-based rechargeable Ni–Zn battery with an outstanding electrochemical performance was demonstrated [32]. Based on this background, a properly engineered Ni/NiO core@shell structure and the interfacial heterojunction between the two can lead to significantly improved ORR and OER performance. Herein, we synthesize a mesoporous Ni/NiO (porous Ni/NiO) nanosheet structure, where both of the constituent phases and their interfaces are purposely fine-tuned in the overall mesoporous structure, aiming at achieving synergistic effects among the components.

Herein, we report an efficient strategy for synthesizing a bifunctional electrocatalyst comprising porous Ni/NiO nanosheets, where the catalyst exhibits outstanding electrocatalytic stability with overpotential of 1.49 V (10 mA cm^−2^) and half-wave potential of 0.76 V in the OER and ORR, respectively. The porous Ni/NiO nanosheet-based Zn–air batteries show long-time cycling stability and high initial charging–discharging transfer efficiency (57.1%) compared with the congeners employing Pt/C-RuO_2_ [33, 34]. The solid-like batteries exhibit the desired mechanical flexibility and overall good electrochemical performance, revealing their potential applications in future flexible devices.

## Experimental Section

### Material Preparation

#### Synthesis of Ni/NiO Nanosheets

In a typical experiment, 0.005 mol NiCl_2_·6H_2_O, 0.1 mol NaOH, and 0.08 mol NaH_2_PO_2_·H_2_O were dissolved in 60 mL deionized (DI) water under vigorous stirring for 2 h. After that, the homogeneous miscible liquid was transferred to a 100-mL Teflon-lined stainless-steel autoclave, which was placed in an oven at 130 °C for 12 h. The resultant precursor was rinsed with DI water and ethanol several times. Finally, the dried precursor was annealed at 400 °C under argon atmosphere for 2 h.

#### Synthesis of Porous Ni/NiO Nanosheets

The as-prepared Ni/NiO nanosheets were post-treated with 1.0 M HCl for 24 h. The product was then separated by centrifuging, washed with sufficient deionized water, and dried.

#### Synthesis of NiO Nanosheets

For comparison, NiO nanosheets were prepared by omitting NaH_2_PO_2_·H_2_O from the above process.

### Material Characterization

Scanning electron microscopy (SEM, Hitachi S-4800) and transmission electron microscopy (TEM, Tecnai G2 F30, FEI) were employed to study the microstructure of the samples. An X-ray diffractometer (XRD; X’pert Pro Philips with Cu-K*α* radiation) was used to identify the crystal structure and phases of the samples. The chemical states and bonding characteristics were analyzed by X-ray photoelectron spectroscopy (XPS; Kratos AXIS Ultra) and Raman spectroscopy (Jobin–Yvon LabRam HR80). The Brunauer–Emmett–Teller (BET) surface area and pore size were measured by using a Micrometrics ASAP 2020 V403 instrument. Fourier transform of the extended X-ray absorption fine structure (EXAFS) spectra were collected at room temperature in the vicinity of the Ni K-edge at the X-ray absorption fine structure for catalysis (XAFCA) beamline of the Singapore Synchrotron Light Source (SSLS), Singapore. The energy was calibrated using a neodymium foil. The EXAFS data were analyzed by using WINXAS 3.1 code. The data were normalized and transformed from energy space to momentum (*k*) space, the background absorption was removed, and the $\chi$(*k*) function was extracted in the range of 1.5‒10.6 Å^−1^. Fourier transform of $\chi$(*k*) weighted by *k*^3^ (i.e., *k*^3^*x*(*k*) in *R* space) was performed using the Bessel function.

### Electrochemical Measurements

Electrochemical measurements were conducted by using a three-electrode setup and a CHI 760E electrochemical workstation at room temperature (25 ± 0.5 °C). Ag/AgCl and a graphite rod or platinum foil were used as the reference electrode and counter electrode, respectively. The measured potentials (vs. Ag/AgCl) were converted to the RHE and *iR* corrected according to the Nernst equation (*E*_RHE_ = *E*_Ag/AgCl_ + 0.197 + 0.059 pH). For the working electrode, 3 mg of the electrocatalyst was dispersed in 1470 μL of *N,N*-dimethylformamide (DMF) and sonicated for 4 h to form the catalyst ink. Thereafter, the fresh catalyst ink (12.6 μL) was dropped onto a rotating disk working electrode (RDE) at a loading of 0.2 mg cm^−2^ and then dried at room temperature. For comparative purposes, commercially acquired Ir/C and Pt/C (20 wt%) were used as reference catalysts. All the ORR and OER properties were tested in 0.1 M KOH solution at room temperature.

### Assembly of Aqueous Zn–Air Batteries

A liquid Zn–air battery was assembled using the home-made air cathode. The aforementioned electrocatalyst ink was dropped onto carbon fiber paper (CFP) at a loading of 2 mg cm^−2^ and dried at room temperature prior to use as the air cathode. A polished Zn plate was used as the anode, and 6 M KOH or 6 M KOH with 0.2 M zinc acetate was used as the electrolyte for the primary and rechargeable Zn–air batteries, respectively. The battery performance was evaluated using a potentiostat (CHI 760E, CH Instrument Co.) and LAND testing system. For comparison, the performance of the Zn–air battery with 20 wt% Pt/C was also tested under the same conditions.

### Solid-like Zn–Air Batteries

A flexible solid-like Zn–air battery was assembled by employing the porous Ni/NiO on carbon fiber paper (CFP) as the air cathode, a zinc anode, and a polymeric hydrogel electrolyte. The polymeric hydrogel electrolyte was prepared as follows: One gram of polyvinyl alcohol (PVA) powder was dissolved in 10 mL of H_2_O at 95 °C under magnetic stirring for 2 h. When the mixture was transformed into a transparent gel, 1 mL of 18 M KOH was added and stirred until the gel became homogeneous. The helical-structured Zn foil was then inserted into a glass bottle with sufficient PVA glue, and the contents of the glass bottle were then frozen to the solid state. The porous Ni/NiO/CFP was tightly attached to the newly assembled helical structure of Zn/PVA with parafilm.

### Calculation Details

The computational code of the Vienna ab initio simulation package (VASP) was used for the spin-polarized DFT calculations. The standard generalized-gradient approximation (GGA) was adopted for the exchange–correlation potential. The energy cutoff of the plane waves used for expanding the electronic wavefunctions was 400 eV. A Monkhorst–Pack *k*-point mesh of 4 × 4 × 4 was used for the geometry optimization and electronic property calculations. The atomic positions and cell parameters were optimized until the residual force was below 0.01 eV Å^−1^. Because of the strong on-site Coulomb repulsion (strong correlation) between the *d* electrons of the Ni atoms, standard density functional theory (DFT) cannot adequately describe the physical properties of NiO. Based on previous recommendations, we used the DFT + U method with *U* = 6.3 eV and *J* = 1 eV to describe the electronic properties of NiO correctly, where *U* and *J* indicate the on-site screened Coulomb energy and exchange interactions, respectively [35]. To calculate the point *O* defects within the periodic boundary conditions, we adopted a Ni_32_O_32_ supercell that is compatible with the type-II antiferromagnetic structure of NiO. In type-II antiferromagnetic ordering, the spin moments of Ni atoms in the same (111) plane are ferromagnetically arranged, and the orientations of the spin moments between adjacent planes are alternately arranged [36]. Ni/NiO heterojunctions were fabricated according to the finite strain theory; the best matching interface follows the orientation relationship of Ni (111)//NiO (111) with an interfacial strain of 2.6%.

## Results and Discussion

**Fig. 1:**
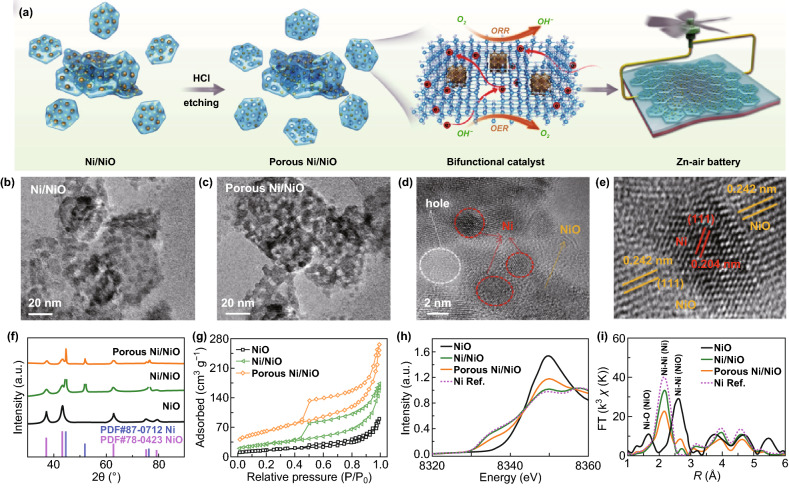
**a** Schematic diagram showing the formation of porous Ni/NiO nanosheets, with excellent OER and ORR performance, and the use of the porous Ni/NiO nanosheets as a highly efficient air electrode for Zn–air batteries. TEM images of **b** Ni/NiO, **c** porous Ni/NiO nanosheets, **d** and **e** high-resolution TEM images of porous Ni/NiO nanosheets. **f** XRD patterns and **g** BET data for NiO, Ni/NiO, and porous Ni/NiO nanosheets. **h** XANES and **i** EXAFS spectra for NiO, Ni/NiO, porous Ni/NiO nanosheets, and Ni reference

The porous Ni/NiO nanosheets were obtained by partially etching the exposed Ni nanoparticles in the Ni/NiO nanosheets. The present aim is to develop efficient bifunctional OER/ORR electrocatalysts by exploiting the synergistic effects of both components, their interfaces, as well as the controlled porous structure (a schematic depiction is presented in Fig. 1a). TEM images of the three products (NiO, Ni/NiO, and porous Ni/NiO nanosheets) are shown in Figs. S1 and 1b, c, respectively. Post-etching treatment with HCl induced the formation of numerous mesopores in the Ni/NiO nanocomposite. Therefore, in the nanosheets, the Ni nanoparticles were implanted in the mesoporous NiO network. To further reveal the crystal structure of both Ni and NiO, high-resolution TEM (HRTEM) studies were conducted, which confirmed the presence of the remaining Ni and mesoporous NiO matrix, as shown in Fig. 1d. There was a clear contrast between the two crystalline phases, as shown in Fig. 1e, where lattice fringes with interplanar distances of ≈ 2.42 and ≈ 2.04 Å were observed for the NiO (111) and Ni (111) facets, respectively, further confirming the development of the Ni/NiO phase in the nanosheets [37, 38]. The XRD patterns of the three products shown in Figs. 1f and S2 indicate that the diffraction peaks are consistent with cubic Ni (JCPDS No. 87-0712) and cubic NiO (JCPDS No. 78-0423). Notably, after the acid etching treatment, the intensity of the Ni diffraction peaks for the mesoporous Ni/NiO decreased considerably. The combined XRD and TEM results confirm partial etching of the Ni nanoparticles by HCl, resulting in the formation of mesopores in the nanosheets. The porous nature of the nanosheets observed by TEM is also supported by the BET surface area measurement, where the nitrogen adsorption–desorption isotherms of the three samples are shown in Fig. 1g. The data show that porous Ni/NiO possesses the typical features of mesoporous materials, with a measured surface area of 186.8 m^2^ g^−1^ and total pore volume of 0.412 cm^3^ g^−1^, which are much higher than those of Ni/NiO (106.3 m^2^ g^−1^, 0.266 cm^3^ g^−1^) and NiO (64.1 m^2^ g^−1^, 0.167 cm^3^ g^−1^). Moreover, the average size of these mesopores was estimated to be 4 nm for the porous Ni/NiO nanosheets (Fig. S3), which is consistent with the HRTEM results presented above.

To verify the local atomic structures of NiO, Ni/NiO, and the porous Ni/NiO nanosheets, the synchrotron X-ray absorption near edge spectra (XANES) were acquired. Figure 1h presents the normalized XANES data for both Ni/NiO and porous Ni/NiO, as compared to those of Ni foil and NiO. The absorption edge of Ni/NiO and porous Ni/NiO was intermediate between the Ni and NiO edges, indicating the mixed oxidation states of Ni (Ni^0^ and Ni^2+^) [39]. Notably, the absorption edge of the Ni/NiO nanosheets was similar to that of Ni, while that of the porous Ni/NiO nanosheets shifted toward that of NiO owing to the decreased Ni content. This observation was further confirmed by Fourier transform of the extended EXAFS *κ*^3^*χ* data for the Ni K-edge and the corresponding oscillations (Figs. 1i, S4). Compared to the signal for the Ni/NiO nanosheets, the intensity of the signal of the Ni–Ni bond (2.1 Å) indicates a decrease in the content of metallic Ni, while the scattering caused by the Ni–O (1.6 Å) and Ni–Ni (2.6 Å) bonds corresponds to an increase in the NiO content for the porous Ni/NiO nanosheets [40, 41]. The XANES and EXAFS data therefore confirm the Ni–O coordination structure and a decrease in the content of metallic Ni in porous Ni/NiO, resulting from acid etching.

**Fig. 2:**
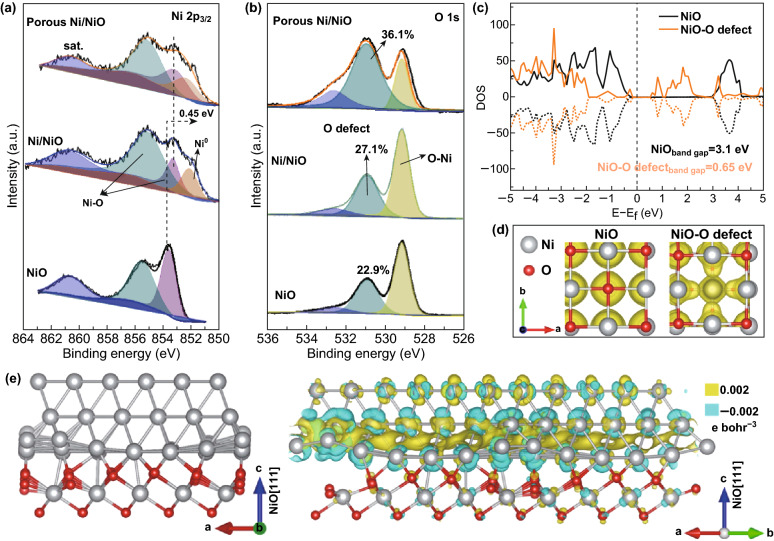
**a** High-resolution Ni 2p and **b** O 2*p* XPS spectra for NiO, Ni/NiO, and porous Ni/NiO nanosheets. **c** Calculated DOS results for NiO with and without O-deficiencies. **d** Calculated partial charge density of NiO with and without O-deficiencies (the isosurface value is 0.001 e/bohr^3^). **e** Structure of the interface formed between Ni (111) and Ni-terminated NiO (111) slabs and the corresponding calculated charge density difference, where yellow and blue represent electron accumulation

XPS analysis was further carried out, and the Ni 2*p* spectra of these three samples are shown in Fig. S5. The fitting results for the Ni 2*p*_3/2_ peaks for each sample are presented in Fig. 2a to reveal their valence states. The peaks at 853.61, 855.60, and 860.71 eV correspond to the Ni–O bond, surface states, and satellite peak of common NiO, respectively [42, 43]. A new peak also appeared at 852.52 eV for both Ni/NiO and porous Ni/NiO, which is related to metallic Ni [44]. Notably, the signal of the Ni–O bond shifted (~ 0.45 eV) toward lower energy for Ni/NiO and porous Ni/NiO, compared to that of pure NiO. This is attributed to the interface and the effect of the O-deficiencies [26, 27]. The O 1*s* spectra (Fig. 2b; Table S1) of all three samples were well fitted to three peaks, where the common two peaks at 529.14 and 532.64 eV reveal the presence of the Ni–O bond and surface adsorbed oxygen, while the peak at 530.96 eV reveals the existence of O-deficiencies in these samples [45]. The percentage of O-deficiencies was highest (36.1%) for the porous Ni/NiO nanosheets, revealing that the acid treatment led to more defects, which may have a positive effect on the electrocatalytic properties [46]. The DFT data provide further evidence to support this claim. As shown in Fig. 2c, pure NiO exhibits semiconductor characteristics with a band gap of 3.10 eV, whereas the band gap of NiO with O-deficiencies was much narrower (0.65 eV), which may contribute to the high conductivity measured for the porous Ni/NiO nanosheets and therefore promote rapid charge transfer during the electrocatalytic process. The increased conductivity of NiO with O-deficiencies was also confirmed by the partial charge density results shown in Fig. 2d. Moreover, Fig. 2e shows the optimized atomic structure of the interface formed between the Ni(111) slab and NiO (111) with terminal Ni, where the charge density difference illustrates the accumulation of electrons at the interface and transfer from NiO to Ni. In the successfully formed porous Ni/NiO nanosheets, the electronic conductivity is effectively improved by the Ni core and O-deficiencies in the nanosheets. The large surface area and mesoporous structure may further increase the number of active sites, and therefore contribute to the highly enhanced electrocatalytic activity for the both OER and ORR, as discussed later.

**Fig. 3:**
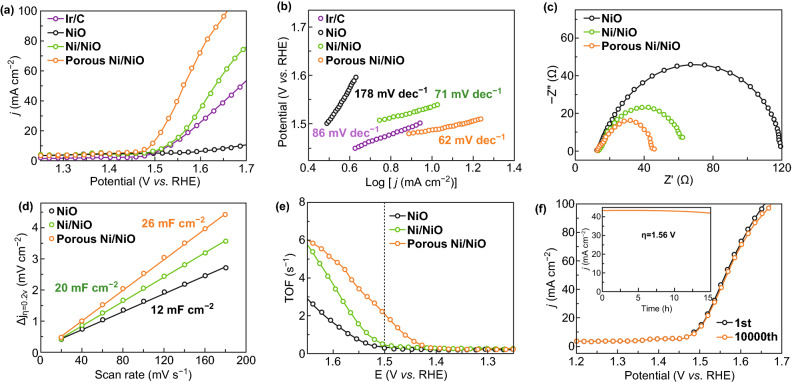
**a** LSV polarization curves, **b** Tafel plots, **c** EIS, **d** ECSA, and **e** TOF data for NiO, Ni/NiO, and porous Ni/NiO catalysts. **f** LSV polarization curves before and after 10,000 CV cycles for porous Ni/NiO catalyst. Inset shows the current density dependence as a function of time at *ƞ* = 1.56 V

Firstly, we examined the electrochemical activity of porous Ni/NiO for the OER, in comparison with that of NiO and the Ni/NiO nanosheets; the experiments were performed in 0.1 M KOH solution at a scan rate of 2 mV s^−1^. All of the potentials were referred to the reversible hydrogen electrode (RHE) and were *iR* corrected. As shown in Figs. 3a, b, and S6, the porous Ni/NiO nanosheets exhibited excellent OER activity with the smallest overpotential of 1.49 V and a Tafel slope of 62 mV dec^−1^, compared with corresponding values of 1.53 V and 71 mV dec^−1^ for the Ni/NiO nanosheets and 1.69 V and 178 mV dec^−1^ for the NiO nanosheets, respectively. Notably, the measured overpotential of 1.49 V is smaller than that of Ir/C (1.53 V), indicating that the porous Ni/NiO nanosheets are an outstanding alternative to commercial Ir/C. The electrochemical impedance spectra (EIS), electrochemically active surface area (ECSA), and turnover frequency (TOF) were measured to gain insight into the OER reaction kinetics and effective active sites of the samples. The Nyquist plots shown in Fig. 3c indicate that porous Ni/NiO exhibited the lowest charge transfer resistance (*R*_ct_) of 46 Ω, where the measured *R*_ct_ values were 63 Ω for Ni/NiO and 118 Ω for NiO, suggesting higher conductivity and faster electron transfer for the porous Ni/NiO catalyst during the OER process. The ECSA represented by the double-layer capacitance (*C*_dl_) shown in Figs. 3d and S7 was 26 mF cm^−2^ for the porous Ni/NiO, which was larger than that of Ni/NiO (20 mF cm^−2^) and twofold higher than that of NiO nanosheets (12 mF cm^−2^), revealing the largest exposed active sites for porous Ni/NiO [47]. The TOF values measured at a potential of 1.5 V were 0.31 (NiO), 0.52 (Ni/NiO), and 2.15 (porous Ni/NiO) O_2_/s, as shown in Fig. 3e, demonstrating that the porous Ni/NiO nanosheets possess more active sites [48]. Stability is also an important parameter in the development of the electrocatalysts. As shown in Fig. 3f, the LSV curve of the porous Ni/NiO nanosheets after 10,000 potential cycles almost overlapped with the initial curve, confirming the outstanding stability of this catalyst against degradation in the accelerated OER process.

**Fig. 4:**
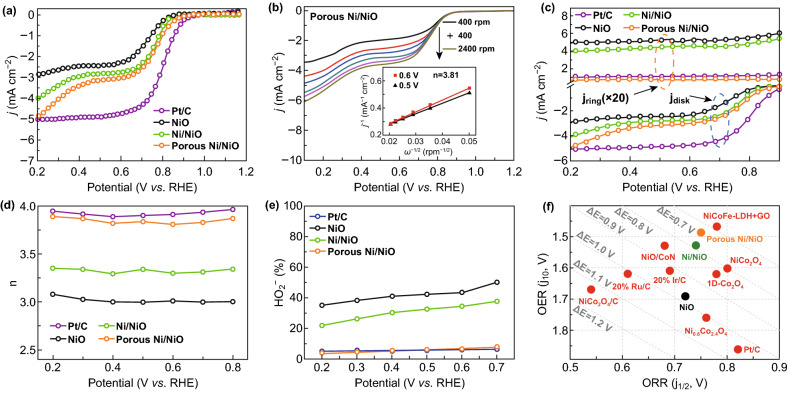
**a** ORR curves for Pt/C, NiO, Ni/NiO, and porous Ni/NiO electrocatalysts. **b** ORR curves for porous Ni/NiO measured at different speeds; *K*–*L* plots obtained at different potentials are shown in the inset. **c** Disk and ring currents on RRDE with a ring potential of 1.5 V (vs. RHE). **d** Calculated *n* at different potentials, and **e** peroxide percentage for Pt/C, NiO, Ni/NiO, and porous Ni/NiO catalysts. **f** ORR and OER bifunctional activity of catalysts in this work compared with representative electrocatalysts in references (Δ*E* represents the typical difference values)

Rotating disk electrode (RDE) measurements were further employed to evaluate the ORR activity of the catalysts in O_2_-saturated 0.1 M KOH solution at a rotating speed of 1600 rpm. From Figs. 4a and S8, S9, the onset potential of the porous Ni/NiO nanosheets (defined by the tangent line where the current density increases rapidly and intersects with the horizontal line) was 0.87 V (~ 0.90 V for Pt/C), with a half-wave potential (*E*_1/2_) of 0.75 V (~ 0.79 V for Pt/C), limiting current density (*j*_L_) of 4.9 mA cm^−2^ (~ 5.0 mA cm^−2^ for Pt/C) (0.2 V vs. reversible hydrogen electrode (RHE)), and kinetic current (*j*_k_) of 18 mA cm^‒2^ (~ 19 mA cm^−2^ for Pt/C) (0.6 V versus RHE), revealing the outstanding ORR performance of the Ni/NiO nanosheets. The number of electrons transferred per O_2_ (*n*), calculated from the Koutecky–Levich (*K*–*L*) plots, was 3.81 for the porous Ni/NiO nanosheets (inset of Figs. 4b, S10), suggesting that the porous Ni/NiO nanosheets favor a nearly four-electron pathway at a relatively low overpotential than other catalysts [49]. The ORR path of the catalysts was further verified by rotating ring-disk electrode measurements (RRDE), and the results are shown in Fig. 4c. The calculated *n* was also above 3.81 (Fig. 4d), and the yield of HO_2_^−^ was below 7.5% (Fig. 4e) for the porous Ni/NiO nanosheets in the voltage region of 0.2–0.7 V, where the values are superior to those of Ni/NiO (*n*: 3.29 and HO_2_^−^: 36.4%) and NiO (*n*: 2.98 and HO_2_^−^: 50.2%). These values are very close to those of the Pt/C catalyst (*n*: 3.86 and HO_2_^−^: 6.3%), clearly demonstrating that the porous Ni/NiO nanosheets can efficiently induce the generation of oxygen at a relatively low overpotential. In general, the overall oxygen electrocatalytic activity and reversibility is estimated from the difference for the OER and ORR (*E *= *E*_*j*=10_ − *E*_1/2_), where a lower *E* value is correlated with better bifunctional activity [50]. Figure 4f shows that the *E* value of 0.75 V for the porous Ni/NiO nanosheets is smaller than those reported for NiO-based or Ni-based bifunctional catalysts (Table S2), further revealing that the porous Ni/NiO nanosheets are an efficient bifunctional OER/ORR electrocatalyst. More importantly, the electrocatalytic stability of the porous Ni/NiO nanosheets for both the ORR and OER was evaluated. The *i*–*t* data in Fig. S11 show 97% and 94% retention for the porous Ni/NiO nanosheets after 15 h for the OER and ORR, which indicates that the Ni/NiO nanosheets are more stable than the Ir/C and Pt/C catalysts, respectively. The XRD, XPS, and TEM data shown in Figs. S12 and S13 indicate that the porous Ni/NiO nanosheets show outstanding stability.

**Fig. 5:**
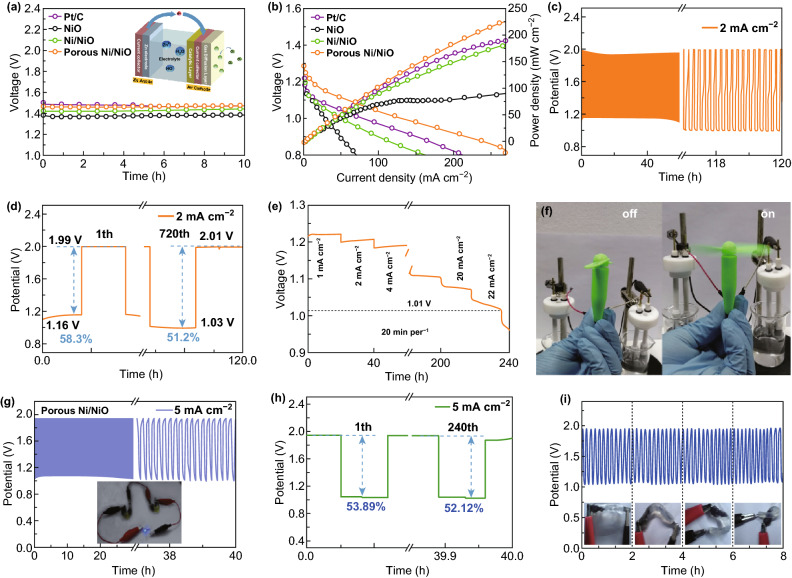
**a**, **b** Open-circuit potential, discharge curves, and power density curves for Pt/C, NiO, Ni/NiO, and porous Ni/NiO. Inset shows schematic diagram for solution Zn–air battery. **c**, **d** Long-term discharge–charge cycling performance at 2 mA cm^−2^ for porous Ni/NiO-based battery. **e** Discharge curves of porous Ni/NiO-based battery at different current densities. **f** Air fan powered by two porous Ni/NiO-based Zn–air batteries in series. **g**, **h** Long-term discharge–charge cycling performance for solid-like Zn–air battery at 5 mA cm^−2^, and **i** performance of porous Ni/NiO-based battery under bending at different angles at 2-h intervals

The remarkable bifunctional electrocatalytic activity of the porous Ni/NiO nanosheets may be attributed to the synergistic effects among the metallic Ni core, O-deficiencies in NiO, the interface between Ni and NiO, and the mesoporous structure. Firstly, the metallic Ni nanoparticles and the O-deficiencies in NiO play an important role in improving charge transport in the OER/ORR processes. Secondly, the conductive electrons can be easily transferred between the two phases owing to electron accumulation at the interface, which enhances the OER/ORR activity of the catalyst [51]. Finally, the mesoporous structure increases the number of exposed active sites, leading to higher electrochemical activity. Given the superior bifunctional activity of the porous Ni/NiO nanosheets discussed above, we assembled a rechargeable Zn–air battery to demonstrate the applicability of the nanosheets in energy storage and transformation. The diagram of the Zn–air battery presented in the inset of Fig. 5a shows three components: the porous Ni/NiO nanosheets as the air cathode, Zn plate as the anode, and 6.0 M KOH + 0.2 M zinc acetate as the electrolyte. The open-circuit potential (OCP) of the porous Ni/NiO nanosheet-based Zn–air battery shown in Fig. 5a is 1.47 V, which is comparable to that of the Pt/C-based battery with an OCP of 1.48 V. This is indeed larger than the values of 1.38 V for NiO and 1.43 V for Ni/NiO (Fig. S14). As shown in Figs. 5b and S15, a maximum power density of 225 mW cm^−2^ was obtained for the porous Ni/NiO nanosheets-based Zn–air battery, whereas that for the Pt/C-based battery was only 185 mW cm^−2^; the value for the Ni/NiO nanosheets is also larger than that of other recently reported systems (Table S3). To confirm its stability, we cycled the air cathode at 2 mA cm^−2^, where the duration of each cycle was 10 min. As shown in Fig. 5c, d, the porous Ni/NiO-based Zn–air battery had a long cycle life over 720 cycles (about 540 cycles for the Ni/NiO-based battery as shown in Fig. S16), where the voltaic efficiency decreased from 58.3% (the difference in the charging–discharging potential was 0.83 V at the first cycle) to 51.2% (the difference in the charging–discharging potential was 0.98 V at the 720th cycle). The results presented in Fig. 5e indicate that the Zn–air battery exhibited better discharging stability with stable performance as the current density was increased over the range of 1 to 22 mA cm^−2^. The specific capacity of the porous Ni/NiO-based Zn–air battery was 853 mAh g_Zn_^−1^ with respect to the consumption of Zn, measured at 20 mA cm^−2^, as shown in Fig. S17, which is much larger than that of 20 wt% Pt/C + IrO_2_ (479 mAh g_Zn_^−1^) [52]. Importantly, as shown in Figs. 5f and S18, two such Zn–air batteries in series could power an air fan and light-emitting diodes, revealing the potential of the Ni/NiO nanosheets for practical applications.

Moreover, we assembled a flexible solid-like Zn–air battery, where porous Ni/NiO was used as the air cathode, a zinc sheet was used as the anode, and polymeric hydrogel was used as the electrolyte. As shown in Fig. 5g, h, this solid-like Zn–air battery exhibited a long cycle life of over 240 cycles with a small decrease in the voltaic efficiency from 53.89% (the difference in the charging–discharging potential was 0.90 V at the first cycle) to 52.12% (the difference in the charging–discharging potential was 0.87 V at the 240th cycle). The OCP of this battery was 1.38 V, and two of these batteries in series could power a blue LED (~ 3 V), as shown in the inset of Figs. 5g and S19. Further, as shown in Fig. S19b–d, the solid-like Zn–air battery showed no major performance decay upon bending and exhibited cycling stability at different bending angles when measured at 2-h intervals (Fig. 5i), implying its potential applicability in future flexible electronics and wearable devices.

## Conclusions

We developed a highly efficient electrocatalyst based on mesoporous Ni/NiO nanosheets, which were synthesized by a scalable process of hydrothermal growth and post-acid etching. The oxygen electrocatalyst is designed to consist of Ni nanoparticles purposely interpenetrated into mesoporous NiO nanosheets (porous Ni/NiO), with well-established pore channels for charge transport at the interface between the nanosheets, oxygen deficiencies at the pore edges (giving rise to enhanced electrical conductivity), and an overall mesoporous structure, leading to a higher surface area and therefore effective exposure of the active sites. Accordingly, the nanosheets show outstanding electrochemical performance and catalytic activity for both the ORR and OER. The application potential was demonstrated in air cathodes for rechargeable and flexible Zn–air batteries. The successful development of the mesoporous Ni/NiO nanosheets provides a new pathway for achieving non-noble metal-based bifunctional oxygen catalysts and air cathodes for Zn–air batteries.

## Electronic supplementary material

Below is the link to the electronic supplementary material.

Supplementary material 1 (PDF 1223 kb)
