# Supplementary material for: Bifunctional Oxygen Electrocatalyst of Mesoporous Ni/NiO Nanosheets for Flexible Rechargeable Zn–Air Batteries
Source: Nanomicro Lett. 2020 Mar 9;12:68. doi: 10.1007/s40820-020-0406-6 (PMC7770935; doi:10.1007/s40820-020-0406-6)
Supplement: Supplementary file 1 — Supplementary material 1 (PDF 1223 kb) [file 40820_2020_406_MOESM1_ESM.pdf]

Supporting Information for

Bifunctional Oxygen Electrocatalyst of Mesoporous Ni/NiO Nanosheets for Flexible Rechargeable Zn–air Batteries

Peitao Liu[1], Jiaqi Ran[1], Baorui Xia[1], Shibo Xi[2], Daqiang Gao[1, *], John Wang[3]

[1]Key Laboratory for Magnetism and Magnetic Materials of MOE, Key Laboratory of Special Function Materials and Structure Design of MOE, Lanzhou University, Lanzhou 730000, People's Republic of China

[2]Institute of Chemical and Engineering Sciences, A*STAR, 1 Pesek Road, Jurong Island, 627833, Singapore

[3]Department of Material Science and Engineering, National University of Singapore, Engineering Drive 3, 117575, Singapore

*Corresponding author. E-mail: gaodq@lzu.edu.cn (D. Q. Gao)

S1 Calculation of Tafel Slope for OER

The specific capacity was calculated by Eq. S1:

$$\eta = a + b * \log j \qquad \text{(S1)}$$

Where η denotes the overpotential, b denotes the Tafel slope, j denotes the current density. The onset potentials were determined based on the beginning of the linear region in Tafel plots.

S2 Calculation of Overpotential for OER

$$\eta = E_{RHE} - 1.23 \qquad \text{(S2)}$$

S3 Calculation of Electrochemically Active Surface Areas for OER

The double-layer capacitance (C_{dl}) of the samples can be determined from the cyclic voltammograms, which is expected to be linearly proportional to the effective surface area. CV measurements performed in the region of 0.1–0.3 V could be mostly considered as the double-layer capacitive behavior. The double-layer capacitance is estimated by plotting the ΔJ at 0.2 V against the scan rate, where the slope is twice of

C_{dl} (Fig. S7).

S4 Calculation of Turnover Frequency (TOF) for OER

TOF values were calculated by assuming that every metal atom is involved in the catalysis:

$$TOF = j \times S/(4 \times F \times n) \quad \text{(S3)}$$

where j (mA cm^{-2}) is the measured current density at η = 400 mV, S is the surface area of the samples electrode, the number 4 means 4 electrons mol^{-1} of O_2, F is Faraday's constant (96,485.3 C mol^{-1}), and n is the moles of coated metal atom on the electrode calculated from m.

The mass activity (A g^{-1}) values were calculated from the catalyst building m (mg cm^{-2}) and the measured current density j (mA cm^{-2}) at η = 400 mV:

$$mass\ activity = j/m \quad \text{(S4)}$$

S5 Calculation of Electron Transferred Number (n) for ORR

The number of electron transfer per O_2 participate in oxygen reduction can be determined by Koutechy–Levich equation:

$$1/j = 1/j_k + 1/B\omega^{1/2} \quad \text{(S5)}$$

where j_k is the kinetic current and ω is the electrode rotating rate. B is determined from the slope of the Koutechy–Levich (K–L) plots based on the Levich equation below:

$$B = 0.2nF(Do_2)^{2/3}\upsilon^{-1/6}Co_2 \quad \text{(S6)}$$

where n represents the transferred electron number per oxygen molecule. F is Faraday constant (F = 96485 C mol^{-1}). Do_2 is the diffusion coefficient of O_2 in 0.1 M KOH (Do_2 = 1.9 × 10^{-5} cm^2 s^{-1}). υ is the kinetic viscosity (υ = 0.01 cm^2 s^{-1}). Co_2 is the bulk concentration of O_2 (Co_2 = 1.2 × 10^{-6} mol cm^{-3}). The constant 0.2 is adopted when the rotation speed is expressed in rpm.

For the Rotating Ring–Disk Electrodes measurements, the $\%HO_2^-$ and transfer number (n) were determined by Eqs. S7 and S8:

$$\%HO_2^- = 200\frac{I_r/N}{I_d + I_r/N} \quad \text{(S7)}$$

$$n = 4\frac{I_d}{I_d + I_r/N} \qquad \text{(S8)}$$

where I_d is disk current, I_r is ring current and N is current collection efficiency of the Pt ring. N was determined to be 0.40.

S6 Calculation of Specific Capacity and Energy Density for Zn–air Batteries

The specific capacity was calculated by Eq. S9:

$$Specific\ Capacity = I \times t/m_{Zn} \qquad \text{(S9)}$$

The energy density was calculated by Eq. S10:

$$Energy\ Density = I \times t \times V/m_{Zn} \qquad \text{(S10)}$$

Where I denotes Current, t denotes the service hours, V denotes the average discharge voltage, and m_{Zn} denotes the weight of consumed zinc.

S7 Supplementary Figures and Tables

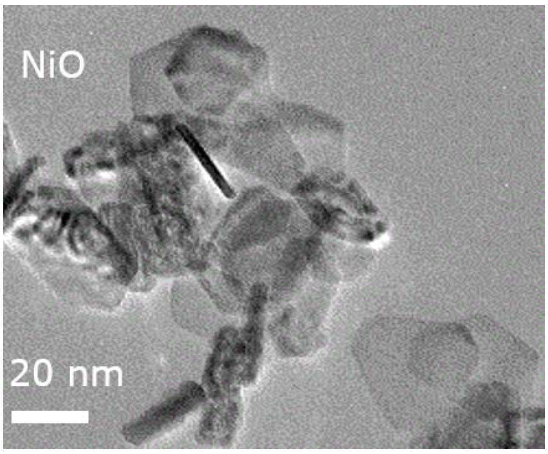

Fig. S1 TEM image for NiO nanosheets

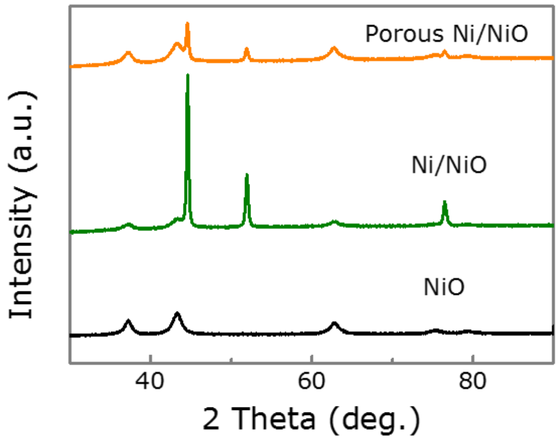

Fig. S2 Original XRD results for NiO, Ni/NiO, and porous Ni/NiO catalysts

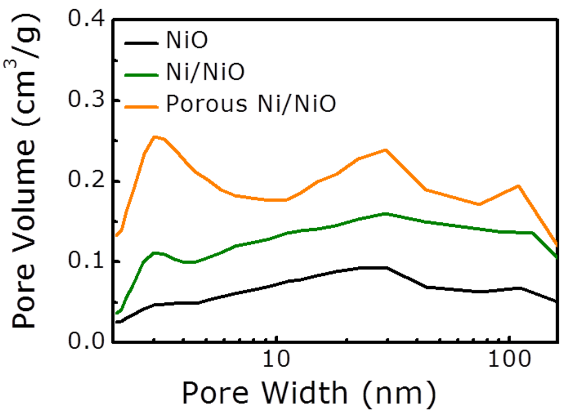

Fig. S3 Pore width distribution curves for NiO, Ni/NiO, and porous Ni/NiO nanosheets

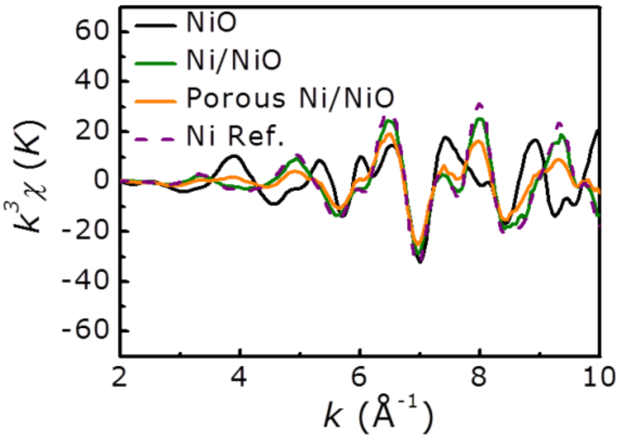

Fig. S4 Fourier-transform of EXAFS k^3x datas at the Ni K-edge and their corresponding oscillations for NiO, Ni/NiO, porous Ni/NiO nanosheets and Ni reference

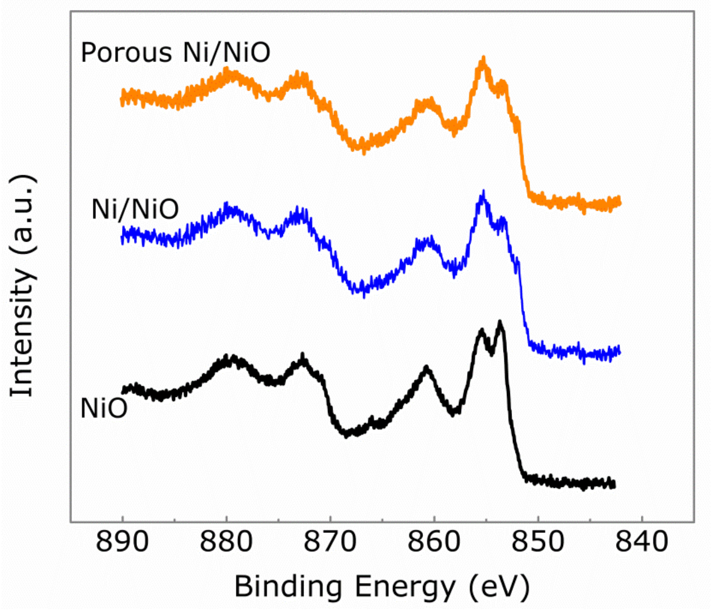

Fig. S5 High-resolution XPS spectra of Ni 2p for NiO, Ni/NiO, and porous Ni/NiO nanosheets

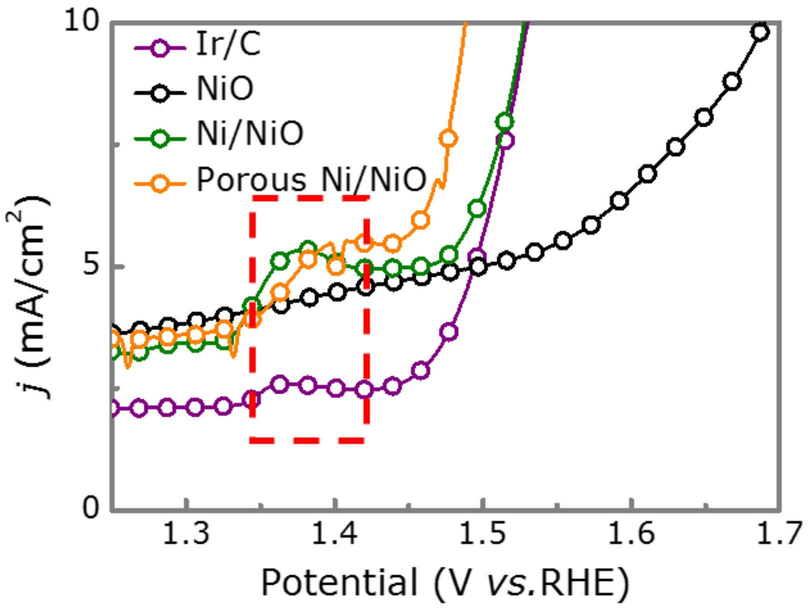

Fig. S6 The LSV polarization curve for Ir/C, NiO, Ni/NiO, and porous Ni/NiO nanosheets

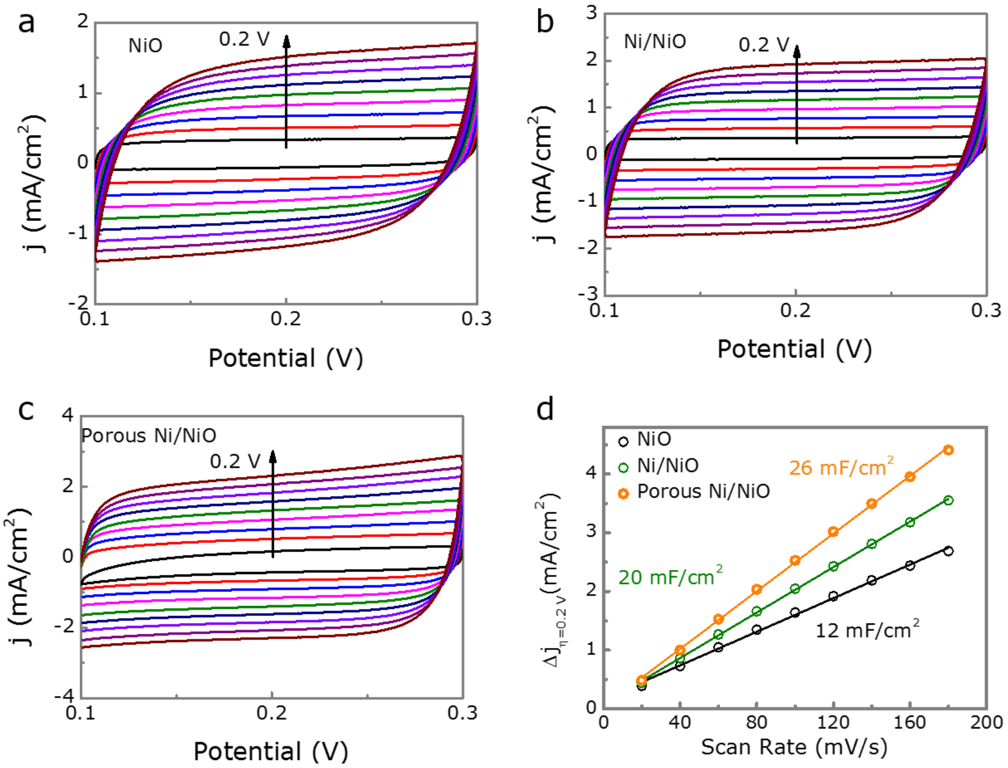

Fig. S7 (**a–c**) CV curves for NiO, Ni/NiO and porous Ni/NiO catalysts measured at different scan rate, respectively. (**d**) The crossponding ECSA results for NiO, Ni/NiO and porous Ni/NiO catalysts

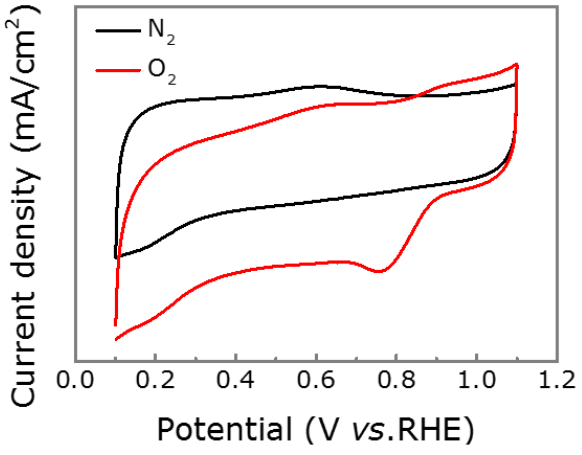

Fig. S8 CV curves for porous Ni/NiO in O_2 and N_2-saturated 0.1 M KOH solution

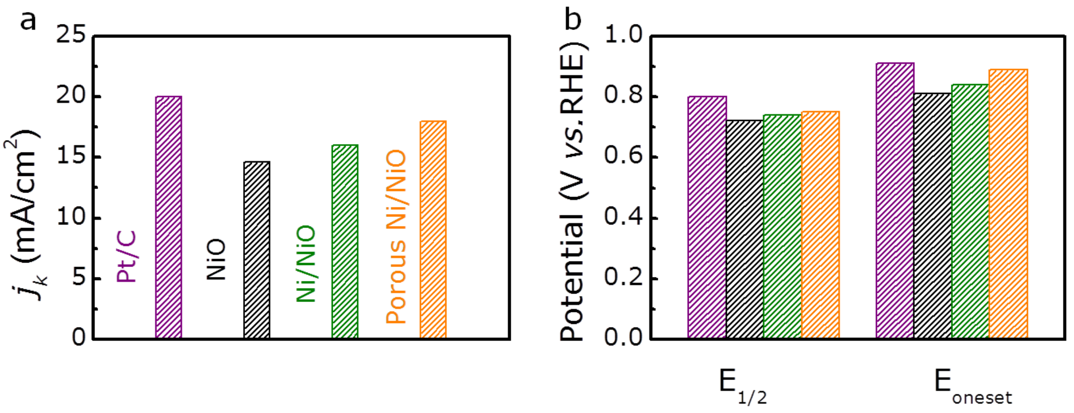

Fig. S9 (**a, b**) The kinetic current, half-wave potential and onset potential for Pt/C, NiO, Ni/NiO, and porous Ni/NiO nanosheets

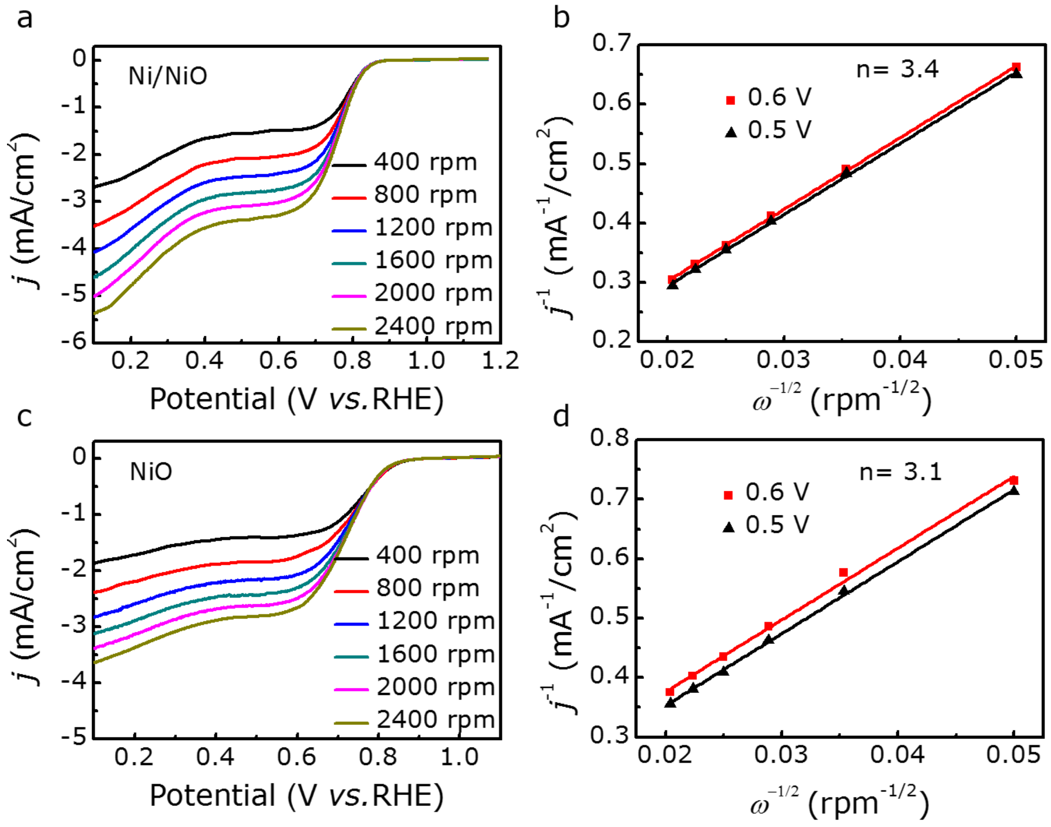

Fig. S10 (**a**) ORR polarization curves for the Ni/NiO at different rotation speeds, and (**b**) the corresponding Koutecky–Levich plots at different potentials. (**c**) ORR polarization curves for the NiO at different rotation speeds, and (**d**) the corresponding Koutecky–Levich plots at different potentials

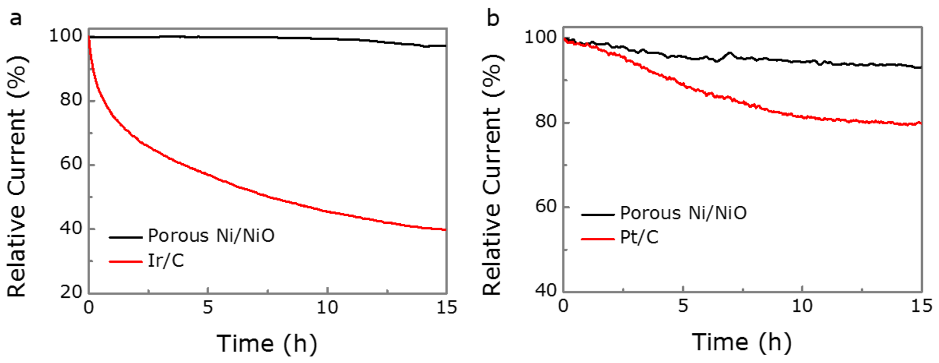

Fig. S11 (**a**) The *i-t* curves of OER for porous Ni/NiO catalyst and Ir/C (20%). (**b**) The *i-t* curves of ORR for porous Ni/NiO catalyst and and Pt/C (20%)

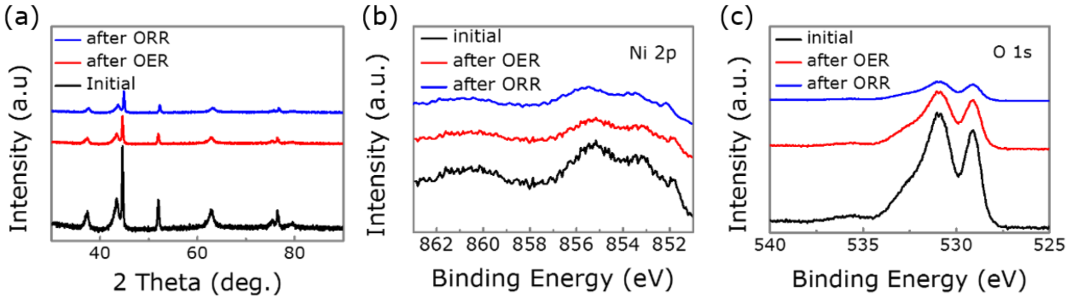

Fig. S12 (**a**) XRD and (**b, c**) XPS results for porous Ni/NiO catalyst for initial and after catalysis

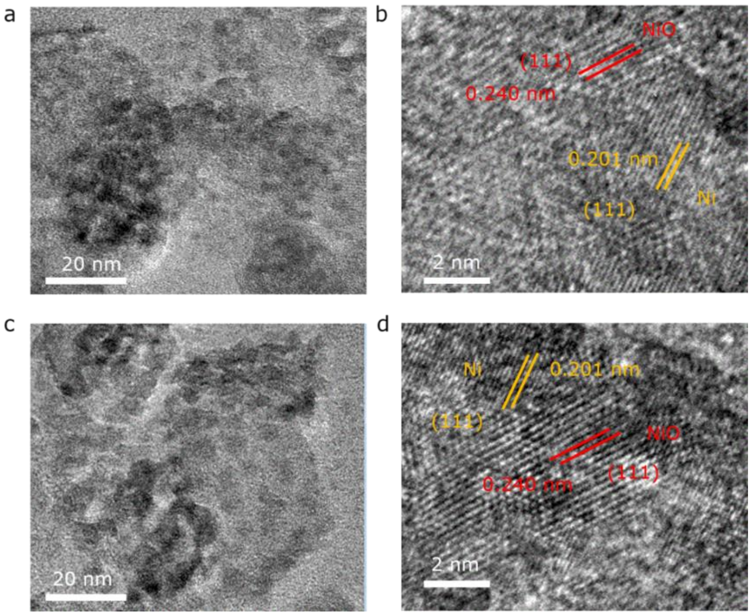

Fig. S13 (**a**) TEM and (**b**) HRTEM images of porous Ni/NiO nanosheets for after OER process. (**c**)TEM and (**d**) HRTEM images of porous Ni/NiO nanosheets for after ORR process

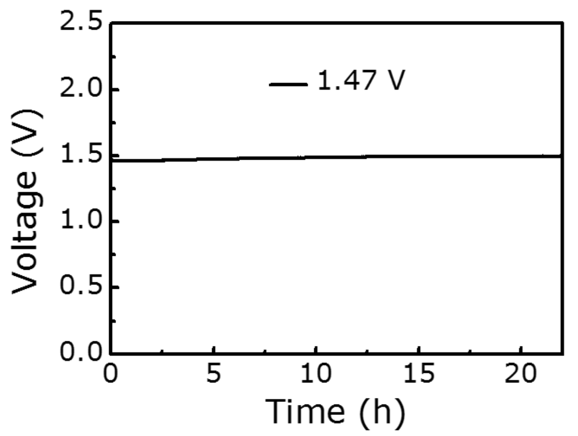

Fig. S14 Open circuit voltage for porous Ni/NiO

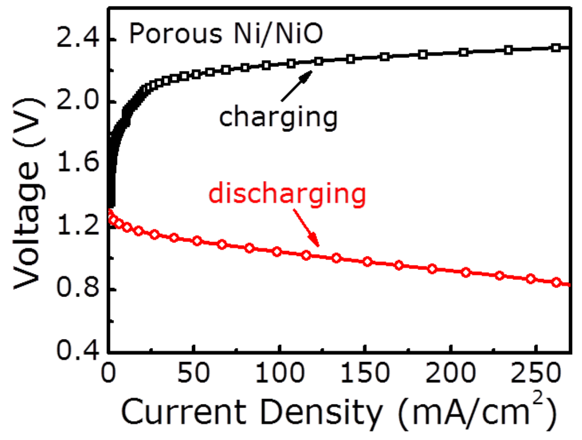

Fig. S15 Charge/discharge polarization curves for porous Ni/NiO

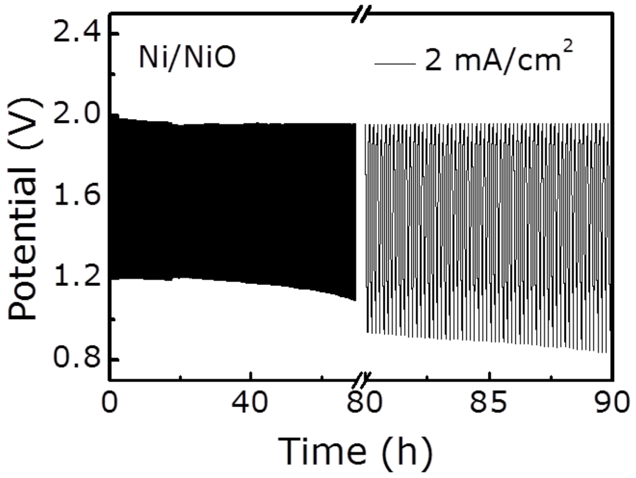

Fig. S16 Long-term discharge–charge cycling performance at a current density of 2 mA cm^{-2} for Ni/NiO

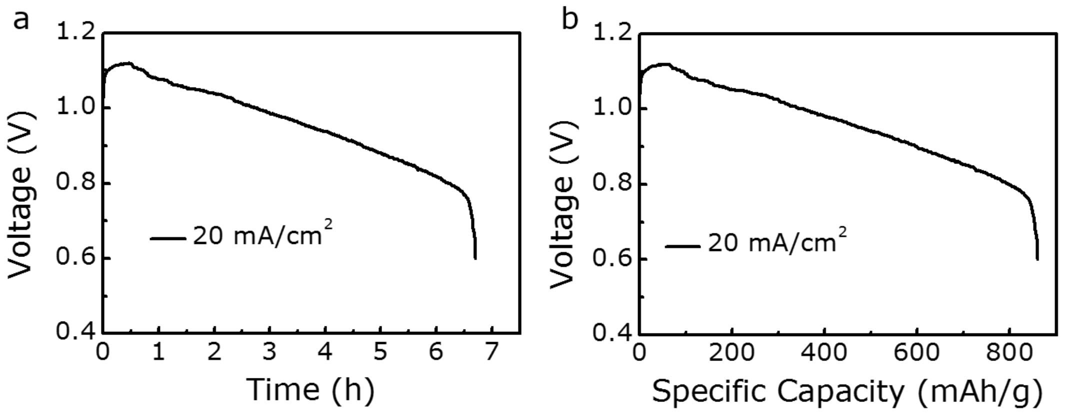

Fig. S17 (**a**) Discharge curve for porous Ni/NiO-based Zn–air battery at 20 mA cm^{-2} and (**b**) the corresponding specific capacity for porous Ni/NiO-based Zn–air battery at 20 mA cm^{-2}

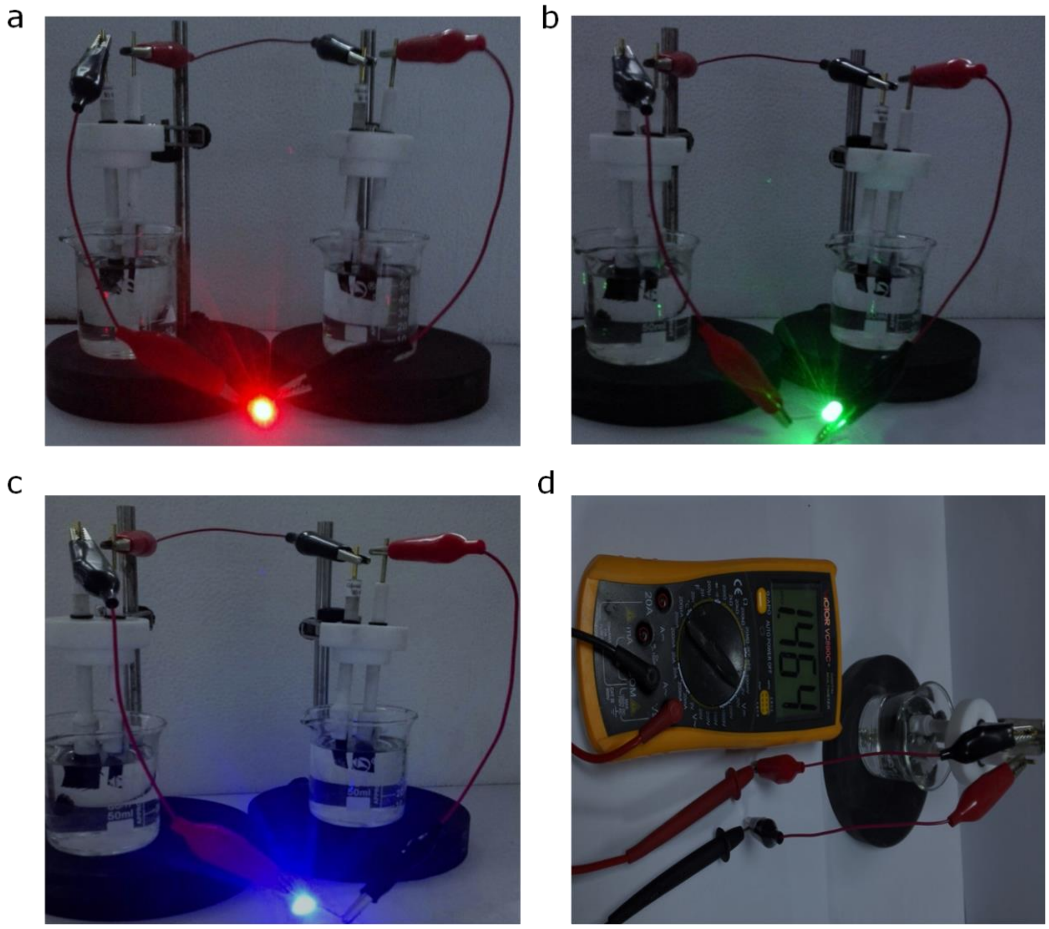

Fig. S18 (**a–c**) Photograph of a red, green and blue LEDs powered by the unpacked two Zn–air batteries with the porous Ni/NiO air-cathode connected in series, respectively. (**d**) Photograph for unpacked Zn–air battery with the porous Ni/NiO air-cathode displays a measured open circuit voltage of ≈1.464 V

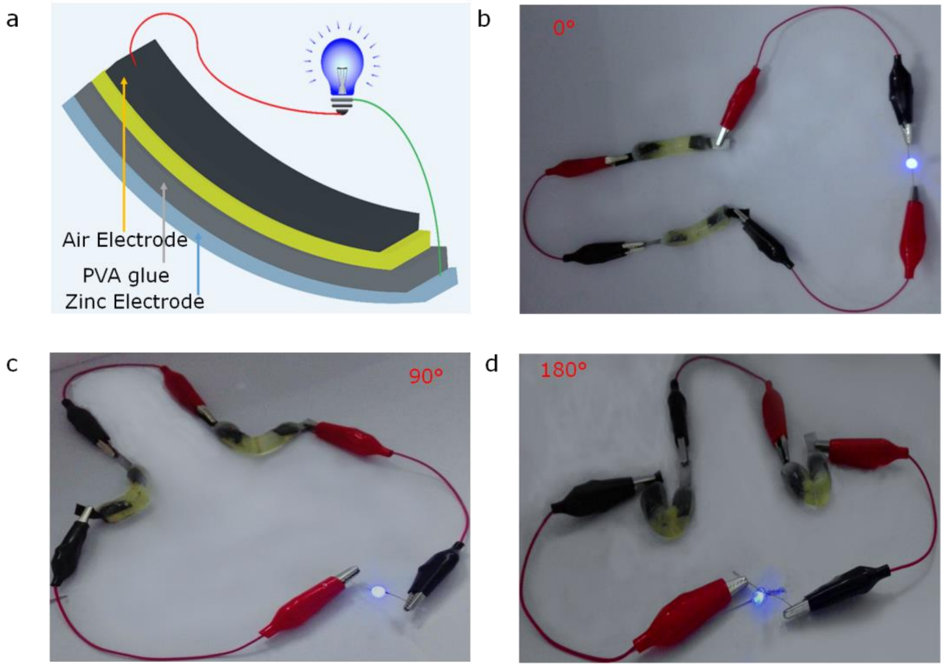

Fig. S19 (**a**) The schematic diagram for solid-like Zn–air battery. (**b–d**) Photograph of a blue LED powered by the unpacked two solid-like Zn–air batteries with the porous Ni/NiO air-cathodes connected in series with different angles

Table S1 Position of O 1s component for NiO, Ni/NiO, and Porous Ni/NiO

Sample	Peak	B.E. (eV)	Area	FWHM (eV)	
NiO	O-Ni	529.13	15013.62	1.23	65.7%
	O defect	530.97	5228.91	1.08	22.9%
	adsorption O	532.59	2591.72	1.20	11.4%
Ni/NiO	O-Ni	529.15	16855.31	0.94	62.3%
	O defect	530.96	7335.97	1.12	27.1%
	adsorption O	532.61	2876.32	1.16	10.6%
Porous Ni/NiO	O-Ni	529.14	9378.14	1.02	35.1%
	O defect	530.96	9656.06	1.08	36.1%
	adsorption O	532.64	7671.54	1.12	28.8%

Table S2 The E ($E = E_{j=10} - E_{1/2}$) value of our work and others NiO-based or Ni-based bifunctional catalysts

Catalysts	$E_{j=10}$ (V) (10 mA cm^{-2})	$E_{1/2}$ (V)	E (V) ($E_{j=10} - E_{1/2}$)	Refs.
NiO	1.69	0.72	0.97	This work
Ni/NiO	1.53	0.74	0.79	This work
porous-Ni/NiO	1.49	0.75	0.74	This work
$NiCo_2O_4/C$	1.67	0.54	1.13	[S1]
20% Ru/C	1.62	0.61	1.01	[S2]
$Ni_{0.6}Co_{2.4}O_6$	1.76	0.76	1.00	[S3]
20% Ir/C	1.61	0.69	0.92	[S2]
Pt/C	1.86	0.82	1.04	[S4]
NiO/CoN	1.53	0.68	0.85	[S5]
1D-$NiCo_2O_4$	1.62	0.78	0.84	[S6]
$NiCo_2S_4$	1.60	0.80	0.80	[S7]
NiCoFe-LDH+GO	1.47	0.78	0.69	[S8]

Table S3 Comparison of the performances of Zn–air batteries of our work and other recently reported catalysts

Catalysts	Electrolyte	Open-circuit potential (V)	Power density (mW cm^{-2})	Refs.
Pt/C	6.0 M KOH	1.48	185	This work
NiO	6.0 M KOH	1.38	90	This work
Ni/NiO	6.0 M KOH	1.43	178	This work
Mesoporous-Ni/NiO	6.0 M KOH	1.47	225	This work
Co-Nx/C NRA	6.0 M KOH	1.42	193.2	[S9]
Meso/Micro-FeCo-N_x-	6.0 M KOH	1.40	150	[S10]
CN	6.0 M KOH	1.46	105	[S11]
C-MOF-C_2-900	6.0 M KOH	1.46	79.6	[S5]
NiO/CoN PINWs	6.0 M KOH + 0.2 M ZnAc	1.45	118.27	[S12]
Co/Co_3O_4@PGS	6.0 M KOH	1.48	82.3	[S13]
Fe_2N@NC	6.0 M KOH + 0.2 M ZnAc	~	250	[S14]
S-treated Fe/N/C	6.0 M KOH + 0.2 M ZnAc	1.49	209	[S15]
S-C_2NA	6.0 M KOH	~	195	[S16]
$Pb_2Ru_2O_{6.5}$	6.0 M KOH + 0.2 M ZnAc	1.44	152	[S17]
Co-N_x-C	6.0 M KOH	1.33	17.4	[S18]
SN-PC-a	6.0 M KOH + 0.2 M ZnAc	1.38	~	[S19]
CoSx@PCN/rGO				

Supplementary References

[S1]D.U. Lee, B.J. Kim, Z. Chen, One-pot synthesis of a mesoporous $NiCo_2O_4$ nanoplatelet and graphene hybrid and its oxygen reduction and evolution activities as an efficient bi-functional electrocatalyst. J. Mater. Chem. A **1**(15), 4754-4762 (2013). https://doi.org/10.1039/c3ta01402a

[S2]Y. Gorlin, T.F. Jaramillo, A bifunctional nonprecious metal catalyst for oxygen reduction and water oxidation. J. Am. Chem. Soc. **132**(39), 13612-13614 (2010). https://doi.org/10.1021/ja104587v

[S3]T.N. Lambert, J.A. Vigil, S.E. White, D.J. Davis, S.J. Limmer, P.D. Burton, E.N. Coker, T.E. Beechem, M.T. Brumbach, Electrodeposited $Ni_{(x)}Co_{(3-x)}O_4$ nanostructured films as bifunctional oxygen electrocatalysts. Chem. Commun. **51**(46), 9511-9514 (2015). https://doi.org/10.1039/C5CC02262B

[S4]Y. Liu, H. Jiang, Y. Zhu, X. Yang, C. Li, Transition metals (Fe, Co, and Ni) encapsulated in nitrogen-doped carbon nanotubes as bi-functional catalysts for oxygen electrode reactions. J. Mater. Chem. A **4**(5), 1694-1701 (2016). https://doi.org/10.1039/C5TA10551J

[S5]J. Yin, Y. Li, F. Lv, Q. Fan, Y.Q. Zhao, Q. Zhang, W. Wang, F. Cheng, P. Xi, S. Guo, NiO/CoN Porous Nanowires as Efficient Bifunctional Catalysts for Zn–Air Batteries. ACS Nano **11**(2), 2275-2283 (2017). https://doi.org/10.1021/acsnano.7b00417

[S6]M. Prabu, K. Ketpang, S. Shanmugam, Hierarchical nanostructured $NiCo_2O_4$ as an efficient bifunctional non-precious metal catalyst for rechargeable zinc–air batteries. Nanoscale **6**(6), 3173-3181 (2014). https://doi.org/10.1039/c3nr05835b

[S7]X. Han, X. Wu, C. Zhong, Y. Deng, N. Zhao, W. Hu, $NiCo_2S_4$ nanocrystals anchored on nitrogen-doped carbon nanotubes as a highly efficient bifunctional electrocatalyst for rechargeable zinc–air batteries. Nano Energy **31**, 541-550 (2017). https://doi.org/10.1016/j.nanoen.2016.12.008

[S8]D. Zhou, Z. Cai, X. Lei, W. Tian, Y. Bi et al., NiCoFe-layered double hydroxides/N-doped graphene oxide array colloid composite as an efficient bifunctional catalyst for oxygen electrocatalytic reactions. Adv. Energy Mater. **8**(9), 1701905 (2018). https://doi.org/10.1002/aenm.201701905

[S9]I.S. Amiinu, X. Liu, Z. Pu, W. Li, Q. Li et al., From 3D ZIF nanocrystals to Co-N_x/C nanorod array electrocatalysts for ORR, OER, and Zn–air batteries. Adv. Funct. Mater. **28**(5), 1704638-1704635 (2018). https://doi.org/10.1002/adfm.201704638

[S10] S. Li, C. Cheng, X.J. Zhao, J Schmidt, A. Thomas, Active salt/silica-templated 2D mesoporous FeCo-N_x-carbon as bifunctional oxygen electrodes for zinc–air batteries. Angew. Chem. Int. Ed. **57**(7), 1856-1862 (2018).

https://doi.org/10.1002/anie.201710852

[S11] M. Zhang, Q. Dai, H. Zheng, M. Chen, L. Dai, Novel MOF-derived Co@N-C bifunctional catalysts for highly efficient Zn–air batteries and water splitting. Adv. Mater. **30**(10), 1705431 (2018). https://doi.org/10.1002/adma.201705431

[S12] Y. Jiang, Y.-P. Deng, J. Fu, D.U. Lee, R. Liang et al., Interpenetrating triphase cobalt-based nanocomposites as efficient bifunctional oxygen electrocatalysts for long-lasting rechargeable Zn–air batteries. Adv. Energy Mater. **8** (15), 1702900 (2018). https://doi.org/10.1002/aenm.201702900

[S13] Z.-Y. Chen, Y.-N. Li, L.-L. Lei, S.-J. Bao, M.-Q. Wang et al., Investigation of Fe_2N@carbon encapsulated in N-doped graphene-like carbon as a catalyst in sustainable zinc–air batteries. Catal. Sci. Technol. **7**(23), 5670-5676 (2017). https://doi.org/10.1039/C7CY01721A

[S14] Q. Wei, G. Zhang, X. Yang, Y. Fu, G. Yang, N. Chen, W. Chen, S. Sun, Litchi-like porous Fe/N/C spheres with atomically dispersed FeNx promoted by sulfur as highly efficient oxygen electrocatalysts for Zn–air batteries. J. Mater. Chem. A **6**(11), 4605-4610 (2018). https://doi.org/10.1039/C7TA08746B

[S15] S.S. Shinde, C.H. Lee, J.Y. Yu, D.H. Kim, S.U. Lee, J.H. Lee, Hierarchically designed 3D holey C_2N aerogels as bifunctional oxygen electrodes for flexible and rechargeable Zn–air batteries. ACS Nano **12**(1), 596-608 (2018). https://doi.org/10.1021/acsnano.7b07473

[S16] J. Park, M. Risch, G. Nam, M. Park, T.J. Shin et al., Single crystalline pyrochlore nanoparticles with metallic conduction as efficient bi-functional oxygen electrocatalysts for Zn–air batteries. Energy Environ. Sci. **10(**1), 129-136 (2017). https://doi.org/10.1039/C6EE03046G

[S17] C. Tang, B. Wang, H.F. Wang, Q. Zhang, Defect engineering toward atomic Co-N_x-C in hierarchical graphene for rechargeable flexible solid Zn–air batteries. Adv. Mater. **29**(37), 1703185 (2017). https://doi.org/10.1002/adma. 201703185

[S18] H. Lin, D. Chen, C. Lu, C. Zhang, F. Qiu, S. Han, X. Zhuang, Rational synthesis of N/S-doped porous carbons as high efficient electrocatalysts for oxygen reduction reaction and Zn–Air batteries. Electrochim. Acta **266**, 17-26 (2018). https://doi.org/10.1016/j.electacta.2018.02.017

[S19] W. Niu, Z. Li, K. Marcus, L. Zhou, Y. Li, R. Ye, K. Liang, Y. Yang, Surface-modified porous carbon nitride composites as highly efficient electrocatalyst for Zn–air batteries. Adv. Energy Mater. **8**(1), 1701642 (2018). https://doi.org/10.1002/aenm.201701642
